# *Citrus unshiu* peel suppress the metastatic potential of murine melanoma B16F10 cells in vitro and in vivo

**DOI:** 10.1002/ptr.6497

**Published:** 2019-09-04

**Authors:** Eun Ok Choi, Hyesook Lee, Hyun HwangBo, Da Hye Kwon, Min Yeong Kim, Seon Yeong Ji, Su Hyun Hong, Gi‐Young Kim, Cheol Park, Hye‐Jin Hwang, Sung‐Kwon Moon, Seok‐Joong Yun, Wun‐Jae Kim, Yung Hyun Choi

**Affiliations:** ^1^ Anti‐Aging Research Center Dong‐eui University Busan Republic of Korea; ^2^ Department of Biochemistry, College of Korean Medicine Dong‐eui University Busan Republic of Korea; ^3^ Laboratory of Immunobiology, Department of Marine Life Sciences Jeju National University Jeju Republic of Korea; ^4^ Department of Molecular Biology, College of Natural Sciences Dong‐eui University Busan Republic of Korea; ^5^ Department of Food and Nutrition, College of Nursing, Healthcare Sciences & Human Ecology Dong‐eui University Busan Republic of Korea; ^6^ Department of Food and Nutrition, College of Biotechnology & Natural Resource Chung‐Ang University Anseong Republic of Korea; ^7^ Personalized Tumor Engineering Research Center, Department of Urology Chungbuk National University College of Medicine Cheongju Republic of Korea

**Keywords:** apoptosis, B16F10 cells, *Citrus unshiu* (CU), melanoma, metastasis

## Abstract

The peel of *Citrus unshiu* Marcow. fruits (CU) has long been used as a traditional medicine that has therapeutic effects against pathogenic diseases, including asthma, vomiting, dyspepsia, blood circulation disorders, and various types of cancer. In this study, we investigated the effect of CU peel on metastatic melanoma, a highly aggressive skin cancer, in B16F10 melanoma cells, and in B16F10 cells inoculated‐C57BL/6 mice. Our results show that ethanol extracts of CU (EECU) inhibited cell growth and increased the apoptotic cells in B16F10 cells. EECU also stimulated the induction of mitochondria‐mediated intrinsic pathway, with reduced mitochondrial membrane potential and increased generation of intracellular reactive oxygen species. Furthermore, EECU suppressed the migration, invasion, and colony formation of B16F10 cells. In addition, the oral administration of EECU reduced serum lactate dehydrogenase activity without weight loss, hepatotoxicity, nor nephrotoxicity in B16F10 cell‐inoculated mice. Moreover, EECU markedly suppressed lung hypertrophy, the number and expression of metastatic tumor nodules, and the expression of inflammatory tumor necrosis factor‐alpha in lung tissue. In conclusion, our findings suggest that the inhibitory effect of EECU on the metastasis of melanoma indicates that it may be regarded as a potential therapeutic herbal drug for melanoma.

## 1 INTRODUCTION

Melanoma is the deadliest skin cancer of melanocytic origin and is a highly aggressive tumor that can metastasize to any organ, including the lungs, liver, bones, and brain (Gray‐Schopfer, Wellbrock, & Marais, 2007). Metastatic melanoma is one of the most intractable cancers because of its unique ability to metastasize early and its resistance against conventional treatments (Bhatia, Tykodi, & Thompson, 2009). The incidence and mortality of melanoma have been rapidly increasing over the past decades, and the number of cases is growing faster than those any other kind of solid cancer (Ko, 2017). In fact, melanoma has a very poor prognosis, and survival rate remains at less than 5% within 5 years (Chi et al., 2011). Furthermore, the mean overall survival of patients with unresectable metastatic melanoma is less than 1 year (Mellman, Coukos, & Dranoff, 2011). Clinical management of patients with metastatic melanoma has been restrictive for treatment because of the few targeted chemotherapies and contrasted protocol available to them (McQuade et al., 2018). Chemotherapies targeting general mutations have been developed, such as a serine/threonine protein kinase of rapidly accelerated fibrosarcoma (RAF) family, another crucial signaling molecule of RAS family, and phosphatase and tensin homologue. However, these drugs have the weakness of inducing resistance and cause unintended side effects and are not fully responsive (Pal, Hunt, Diamond, Elmets, & Afaq, 2016; Villareal, Sato, Matsuyama, & Isoda, 2018). Therefore, there is a growing need to develop new, effective, and safe treatments for metastatic melanoma. In this respect, phytochemicals are coming into the spotlight due to their low cost, low toxicity, and low hostility as dietary supplements (Pal et al., 2016). Experimentally, numerous medical plants and herbal pharmacologically active constituents have been reported to have anticancer, antimetastatic, antiangiogenic, and proapoptotic effects in in vitro and in vivo studies (Shu, Cheung, Khor, Chen, & Kong, 2010; Teiten, Gaascht, Dicato, & Diederich, 2013). Several medical plants and phytochemicals, including *Allium sativum*, *Panax ginseng*, *Rhus verniciflua*, *Viscum album*, camptochecin, curcumin, and resveratrol, have acceptable clinical evidence that supports their anticancer efficacy (Hosseini & Ghorbani, 2015). Actually, some of the pharmacologically active compounds (i.e., taxol analogues, vinca alkaloids, and podophyllotoxin analogues) isolated from these plants are widely used for chemotherapy for patients with cancer (Saklani & Kutty, 2008).

Among such plants, the peel of *Citrus unshiu* Marcow. fruits (CU) called “Jinpi” in Korea, and “Chenpi” in China, has long been used as a traditional medicine in East Asia for the treatment of asthma, vomiting, dyspepsia, and blood circulation disorders (Park, Hwang, Choi, & Ma, 2018). Recently, CU peel has been shown to have multiple therapeutic effects against obesity (Kang, Song, Lee, Chang, & Lee, 2018), depression (Lim et al., 2018), inflammation (Oh et al., 2012; Park et al., 2013), and viral infection (Suzuki et al., 2005). Moreover, several scientists have reported the anticancer effect of CU. According to the study by Lee et al., (Lee, Lee, Kim, & Kim, 2018), fermented extract of CU peel inhibited the growth of human pancreatic cancer cells via the induction of caspase‐3 cleavage. In 2011, one study reported CU has an antitumor effect through enhancing immune‐mediated cytokines in murine renal carcinoma cells (Lee et al., 2011); Jin et al. (2013) suggested that phytochemicals from CU inhibit cell adhesion and invasion in human breast cancer cells. Our previous studies also clearly demonstrated that water and ethanol extracts of CU peel have anticancer effect via the involvement of reactive oxygen species (ROS)–dependent activation of adenosine monophosphate‐activated kinase in human breast cancer MCF‐7 cells (Kim et al., 2018; Kim et al., 2018). In addition, we have recently reported that CU peel induced ROS‐mediated apoptosis in human breast carcinoma cells (Kim et al., 2018) and human bladder cancer cells (Ahn et al., 2017). Based on these previous studies, it is expected that CU peel will have a positive effect on the prevention of various types of cancer. However, there has been no report on the inhibitory effect of CU peel on the metastasis of melanoma cell in vitro and in vivo. Accordingly, we investigated the effect of CU peel on the metastatic potential of B16F10 cells, known to be malignant melanoma cells that are stable in their metastatic potential, in vitro and in vivo.

## 2 MATERIALS AND METHODS

### 2.1 Preparation of 70% ethanol extract of CU peel

The dried peels of CU (100 g, purchased from Dong‐eui Korean Medical Center, Busan, Republic of Korea) were ground into fine powder and refluxed with 1 L of 70% ethanol solution by sonication for 24 hr. After filtering through a glass filter funnel, the extract was concentrated with a rotary vacuum evaporator (Buchi Labortechnik, Flawil, Switzerland), followed by lyophilization, and then stored at −80°C. The freeze‐dried powder of ethanol extract of CU (EECU) was dissolved in dimethylsulfoxide (Sigma‐Aldrich Chemical Co., St. Louis, MO, USA) to a final concentration of 100 mg/mL, and the stock solution was diluted with a cell culture medium to the desired concentration, prior to use.

### 2.2 In vitro study: B16F10 mouse melanoma cells

#### 2.2.1 Cell culture

Murine melanoma B16F10 cells were purchased from the American Type Culture Collection (Manassas, MD, USA). Cells were cultured at 37°C in 5% CO_2_ humidified incubator in complete media consisting of Dulbecco's modified Eagle's medium supplemented with 10% fetal bovine serum (FBS), 100 U/mL penicillin, and 100 μg/mL streptomycin (all from WelGENE Inc., Daegu, Republic of Korea).

#### 2.2.2 Cell viability

The viability of the cells was assessed by 3‐(4,5‐dimethyl‐2‐thiazolyl)‐2,5‐diphenyltetra ‐zolium bromide (MTT; Invitrogen, Thermo Fisher Scientific Inc., Waltham, MA, USA) assay as previously described (Kim, Bo, et al., 2018). Briefly, B16F10 cells were seeded onto 96‐well plates at a density of 1 × 10^4^ cells/well and incubated for overnight. Thereafter, the cells were treated with the desired concentrations of EECU of 0, 20, 40, 60, 80, and 100 μg/mL for 24 hr, and the cells were then incubated with 50 μg/mL MTT solution for 2 hr. Formazan crystals were dissolved in dimethylsulfoxide, and the absorbance was measured using a microplate reader (VERSA Max, Molecular Device Co., Sunnyvale, CA, USA) at 540 nm. The morphological changes of cells were visualized with phase‐contrast microscope (Axio Aver. A1, Carl Zeiss, Oberkochen, Germany).

#### 2.2.3 Analysis of apoptosis

The alteration of nuclear morphology in apoptotic cells was assessed by cell‐permeable nucleic acid stains, 4′,6′‐diamidino‐2‐phenylindole (DAPI; Sigma‐Aldrich Chemical Co., St. Louis, MO, USA). After 24 hr treatment with EECU of 0, 50, and 100 μg/mL, the cells were harvested and fixed with 4% paraformaldehyde for 10 min at room temperature (RT). The fixed cells were washed twice with phosphate‐buffered saline (PBS) and stained with 1 μg/mL DAPI solution for 10 min, under light‐shielded conditions. The cells were washed twice with PBS, and the fluorescence intensity was observed using a fluorescence microscope (EVOS FL Auto2, Carl Zeiss). The magnitude of apoptosis was measured by flow cytometry using the annexin V‐fluorescein isothiocyanate (FITC) Apoptosis detection kit (BD Biosciences, San Diego, CA, USA). In brief, the cells were washed twice with cold PBS and resuspended in 500 μL binding buffer, according to the manufacturer's protocol. The cells were double‐stained with FITC‐labelled annexin V and propidium iodide (PI) for 20 min in the dark. At least 10,000 cells were acquired for each sample and analyzed using a BD Accuri C6 flow cytometer (BD Biosciences, San Jose, CA, USA).

#### 2.2.4 Mitochondrial membrane potential (Δ*ψ*_*m*_)

To observe the mitochondrial membrane potential (MMP, Δ*ψ*_m_), 5,5′,6,6′‐tetrachloro‐1,1′,3,3′‐tetraethyl‐imidacarbocyanine iodide (JC‐1; Sigma‐Aldrich Chemical Co.) staining was performed. After 24 hr treatment with EECU of 0, 20, 60, and 100 μg/mL, 10 μM JC‐1 was added to the cells for 30 min at 37°C. Subsequently, the cells were washed twice with PBS to remove unbound dye, and at least 10,000 cells were collected for each sample. The amounts of MPP were detected at 488/575 nm using a flow cytometer (BD Biosciences) by following the manufacturer's protocol. A plot of red fluorescence (FL2‐H) from living cells with intact MPP and green fluorescence (FLl‐H) from cells with loss of MPP was recorded.

#### 2.2.5 Intracellular ROS generation

The production of intracellular ROS was measured by flow cytometer using 2′,7′‐dichlorofluorescin diacetate (DCF‐DA; Invitrogen) as previously described (Kim, Choi, et al., 2018). Briefly, B16F10 cells were seeded onto 6‐well plate at a density of 2 × 10^5^ cells and then treated with EECU of 0, 20, 60, and 100 μg/mL. In the last 20 min of treatment, 10 μM DCF‐DA was added to the incubated cells in the dark. Following incubation, the cells were washed twice with PBS, and 10,000 cells were analyzed for the amount of intracellular ROS by BD Accuri C6 software in a flow cytometer (BD Biosciences) at 480/520 nm.

#### 2.2.6 Cell migration and invasion assay

To evaluate cell migration, wound lines in the form of a cross were made by scraping with a plastic 200‐μL pipette tip in confluent cells. After wounding, floating cells were washed out with PBS and were incubated with 1% FBS‐containing medium supplemented with or without EECU for 24 hr. Subsequently, the width of wound healing was photographed under an inverted microscope (Carl Zeiss). In vitro invasive activity was assessed using the Trans‐well chamber system (10 mm diameter, 8 μm pore size with polycarbonate membrane; Corning Costar Corp., Cambridge, MA, USA). B16F10 cells were kept in serum‐free medium for 24 hr. The cells (5 × 10^4^ cells/well) were placed in the upper chamber of trans‐well insert, and at the same time, 10% FBS‐containing complement medium supplemented with EECU of 0, 20, and 40 μg/mL was added into the lower chamber, and then cells were incubated for 24 hr. Cells that invaded through the filter were fixed with 4% paraformaldehyde and stained with hematoxylin and eosin (H&E; Sigma‐Aldrich Chemical Co.). The stained cells were counted and observed under an inverted microscope (Carl Zeiss).

#### 2.2.7 Colony formation assay

After treatment with EECU of 0, 20, and 40 μg/mL for 24 hr, single‐cell suspensions of B16F10 cells were placed onto 6‐well plates. The cells were incubated for 2 weeks until the formation of colonies. After fixation with 3.7% paraformaldehyde, the colonies were stained with 0.1% crystal violet solution (Sigma‐Aldrich Chemical Co.) for 10 min at RT. The stained cells were counted and observed under an inverted microscope (Carl Zeiss).

#### 2.2.8 Analysis of metalloproteinase activity

The cells were treated with EECU of 0, 20, and 40 μg/mL for 24 hr, and then cell culture supernatants were harvested, to measure the activities of metalloproteinase (MMP)–2 and MMP‐9. The activities of MMP‐2 and MMP‐9 were determined using Biotrak Activity Assay system from Amersham Biosciences (Piscataway, NJ, USA), according to the manufacturer's instructions.

#### 2.2.9 Western blot analysis

Total protein was extracted from the cells using the Bradford Protein assay kit (Bio‐Rad Laboratories, Hercules, CA, USA). An equal amount of protein (30 μg per lane) from the samples was separated by denaturing sodium dodecyl sulfate (SDS)–polyacrylamide gel electrophoresis and transferred onto polyvinylidene difluoride membranes (Schleicher & Schuell, Keene, NH, USA). The membranes were blocked with 5% skim milk in Tris‐buffered saline containing 0.1% Triton X‐100 (TBST) for 1 hr and probed with specific primary antibodies at 4°C overnight. B‐cell lymphoma 2 (Bcl‐2; sc‐783), Bcl‐2 like protein 4 (Bax; sc‐493), MMP‐2 (sc‐10736), MMP‐9 (sc‐10737), tissue inhibitors of metalloproteinase (TIMP)–1 (sc‐5538) and TIMP‐2 (sc‐5539), and β‐actin (sc‐1616) antibodies were purchased from Santa Cruz Biotechnology (Santa Cruz, CA, USA). Caspase‐3 (#9662) and poly (ADP‐ribose) polymerase (PARP; #9542) were obtained from Cell Signaling Technology (Danvers, MA, USA). After washing three times with TBST, the membranes were incubated with the appropriate horseradish peroxidase (HRP)–conjugated secondary antibodies for 2 hr (RT). Goat anti‐mouse IgG‐HRP and goat anti‐rabbit IgG‐HRP antibodies were purchased from Santa Cruz Biotechnology. The expression of protein was detected by enhanced chemiluminescence kit (GE Healthcare Life Sciences, Little Chalfont, UK) and visualized by Fusion FX Image system (Vilber Lourmat, Torcy, France).

#### 2.2.10 Reverse transcription‐polymerase chain reaction

Total RNA from B16F10 cells was isolated using TRIzol reagent (Invitrogen), following the protocol of the manufacturer. cDNA was synthesized using AMV Reverse Transcriptase (Amersham Corp.) from 1 μg of total RNA. The polymerase chain reaction (PCR) was carried out using the Mastercycler (Eppendorf, Hamburg, Germany) with the following primers: MMP‐2, (sense) 5′‐CTT CTT CAA GGA CCG GTT CAT‐3′, (antisense) 5′‐GCT GGC TGA GTA GAT CCA GTA‐3′; MMP‐9, (sense) 5′‐TGG GCT ACG TGA CCT ATG ACC AT‐3′, (antisense) 5′‐GCC CAG CCC ACC TCC ACT CCT C‐3′; TIMP‐1, (sense) 5′‐TGG GGA CAC CAG AAG TCA AC‐3′, (antisense) 5′‐TTT TCA GAG CCT TGG AGG AG‐3′; TIMP‐2, (sense) 5′‐GTC AGT GAG AAG GAA GTG GAC TCT‐3′, (antisense) 5′‐ATG TTC TTC TCT GTG ACC CAG TC‐3′; GAPDH, (sense) 5′‐CGG AGT CAA CGG ATT TGG TCG TAT‐3′, and (antisense) 5′‐AGC CTT CTC CAT GGT GGT GAA GAC‐3′. Conditions for the PCR were performed as previously described (Choi et al., 2017).

### 2.3 In vivo study: Antimetastatic activity of EECU in B16F10 cells‐inoculated C57BL/6 mice

#### 2.3.1 Animal and experimental procedures

This study was conducted in accordance with the Guidelines for Animal Experimentation of Dong‐eui Uuniversity, with the approval of the Institutional Animal Care and Use Committee (No. R2017‐004) for the use of animal research. We purchased 44 C57BL/6 mice (male, 8 weeks old) from Samtako (Osan, Korea). After acclimatization for 1 week, 28 mice were injected into the tail vein with 3 × 10^5^ B16F10 cells per 100 μL PBS (JW Pharmaceutical, Seoul, Korea). At the same time, 16 mice were injected in the same area with PBS (Figure 4a). After 1 day of tumor inoculation, B16F10 cell‐injected mice were randomly divided into three groups: the B16 + control group (*n* = 10, 100 μL of distilled water), the B16 + EECU 100 group (*n* = 8, 100 μL of EECU 100 mg/kg/day), and the B16 + EECU 200 group (*n* = 8, 100 μL of EECU 200 mg/kg/day). Eighteen PBS‐injected mice were also randomly divided into two groups: the normal group (*n* = 8, 100 μL of distilled water) and the EECU 200 group (*n* = 8, 200 μL of EECU 200 mg/kg/day). All treatments were administrated orally once per day in the morning for 21 days. Mice were sacrificed at Day 21 after B16F10 melanoma cells injection, and blood was placed in heparinized tubes, centrifuged at 3,000 rpm for 10 min at 4°C, and kept at −80°C for subsequent analysis. After perfusion, organs were immediately surgically excised, including liver, kidney, spleen, lung, and thymus, then weighed, and stored at −80°C.

#### 2.3.2 Biochemical analysis

Plasma alanine aminotransferase (ALT; ab105134) and aspartate aminotransferase (AST; ab105135) were measured with a colorimetric assay, using reagents from Abcam Inc. (Cambridge, UK). Lactate dehydrogenase (LDH; K726) activity and blood urea nitrogen (BUN; ABIN577679) were analyzed using detection kit according to the manufacturer's instructions, which kits were obtained from BioVision, Inc. (Milpitas, CA, USA) and antibodies‐online GmbH (Aachen, Germany), respectively.

#### 2.3.3 Histology and immunohistochemistry

Histological analysis was performed as described previously (Kwon et al., 2018). Lung was fixed in 4% formalin and embedded in paraffin. The sections of 5 μm thickness were cut by microtome (Leica RM2125, Leica Biosystems, Heidelberg, Germany) and were stained with H&E. For immunohistochemistry analysis of the lung tissue, the sections of 5 μm thickness were deparaffinized, rehydrated, cooked in antigen retrieval solution (Abcam, Inc.), and dipped in 3% hydrogen peroxide solution for 30 min. Tumor necrosis factor alpha (TNF‐α; Cat No. ab6671, Abcam, Inc.) antibody was then applied and incubated for 1 hr at RT. After washing, the sections were incubated with secondary antibody (DAKO Corp, Glostrup, Denmark) for 40 min. Immunoreactions were visualized with diaminobenzidine chromogen, and the sections were counterstained with Mayer's hematoxylin (Sigma‐Aldrich Chemical Co.) for 30 s at RT. Images of the sections were photographed with microscope (Carl Zeiss).

### 2.4 Chromatographic analysis

The phytochemical compositions of EECU were analyzed via high‐performance liquid chromatography (HPLC; Agilent 1100 series, Agilent Technologies, San Jose, CA, USA), as previously reported (Kim, Bo, et al., 2018). The column used was an OptimaPak C 18 column (RS Tech Co., Daejeon, Republic of Korea), and ultraviolet spectra, scanning 190 to 400 nm, were recorded for all peaks. Standard samples, including naringin, hesperidin, and nephesperidin, were obtained from Sigma‐Aldrich Chemical Co. and dissolved in methanol at 0.15 to 200 μg/mL. The EECU sample was diluted in methanol at 5 mg/mL. Figure 6 and Table 1 show the chromatograms and quantitative results of the HPLC analysis of standard samples and EECU.

**Table 1:** The quantitative results from the HPLC analysis of EECU for naringin, hesperidin, and neohesperidin.

Number	Compound	UV (nm)	ReT (min)	Linear range (μg/mL)	Regression equation	*r* ^2^	Concentration (μg/mg)
1	Naringin	276	30.32	0.3–100	y = 30.8273x + 16.5279	.999	2.147 ± 0.0217
2	Hesperidin	276	31.72	0.3–100	y = 21.9330x + 11.4494	.999	5.614 ± 0.0197
3	Neohesperidin	276	33.42	0.3–100	y = 19.5115x + 9.5025	.999	0.001 ± 0.0122

Abbreviations: EECU, ethanol extract of *Citrus unshiu* peel; HPLC, high‐performance liquid chromatography; ReT, retention time; r^2^, correlation coefficient; UV, ultraviolet.

### 2.5 Statistical analysis

All experiments were performed at least three times. Data were analyzed using GraphPad Prism software (version 5.03; GraphPad Software, Inc., La Jolla, CA, USA) and expressed as the mean ± *SD*. Differences between groups were assessed using analysis of variance followed by ANOVA‐Tukey's post hoc test, and *p* < .05 was considered to indicate a statistically significant difference.

## 3 RESULTS

### 3.1 EECU induced apoptotic cell death in B16F10 melanoma cells

**Figure 1:**
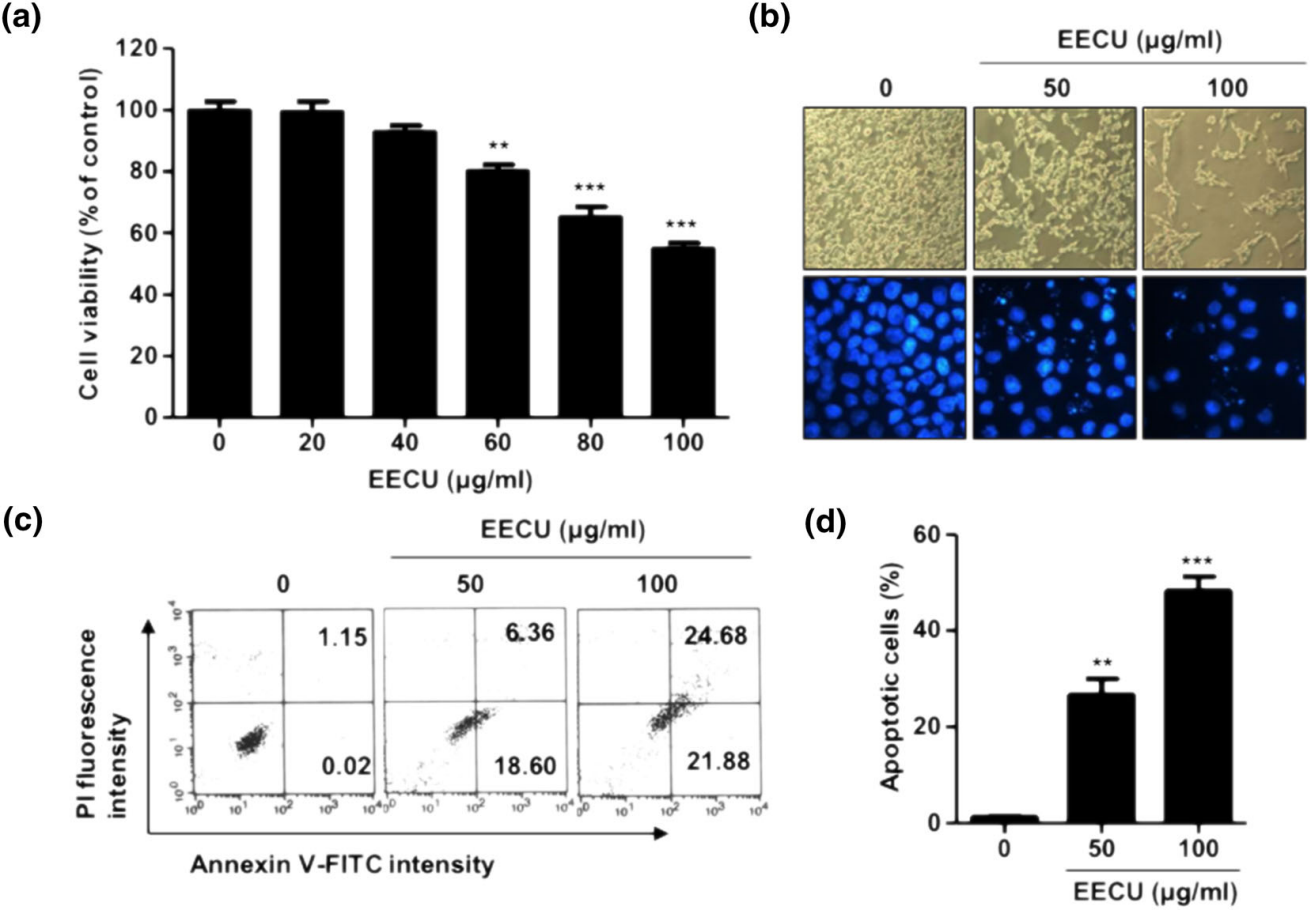
Ethanol extracts of *Citrus unshiu* Marcow. fruits (EECU) induced apoptotic cell death in B16F10 melanoma cells. (a) Cells were incubated with EECU of 20 to 100 μg/mL for 24 hr. The cell viability was assessed by MTT assay. Data are expressed as the mean ± *SD* (*n* = 3). The statistical analyses were conducted using analysis of variance (ANOVA‐Tukey's post hoc test) between groups. ^**^ *p* < .01 and _***_ *p* < .001 when compared with control. (b; top) The morphological change of B16F10 cells treated with EECU for 24 hr was observed under a microscope at 40× magnification. (Bottom) The nuclear morphological change was observed using DAPI staining, and was photographed under a fluorescence microscope at 400× magnification. (c) Apoptosis of B16F10 cells treated with EECU was measured by flow cytometric analysis using annexin V‐fluorescein isothiocyanate (V‐FITC) and propidium iodide (PI). The percentage of annexin V^+^/PI^+^ cells in the top and annexin V^+^/PI^−^ cells in the bottom right quadrant are indicated. Each point represents the mean of three independent experiments. (d) The percentage of apoptotic cells are shown in the bar diagram as the mean ± *SD* (*n* = 3). The statistical analyses were conducted using analysis of variance (ANOVA‐Tukey's post hoc test) between groups. ^**^ *p* < .01 and ^***^ *p* < .001 when compared with control [Colour figure can be viewed at http://wileyonlinelibrary.com]

In order to evaluate the cytotoxicity of EECU, B16F10 cells were incubated with EECU of 0–100 μg/mL for 24 hr, and cell viability was assessed by MTT assay. Figure 1a shows that EECU markedly reduced B16F10 cells viability in concentration of over 60 μg/mL in a dose‐dependent manner (60 μg/mL EECU: 80.33%, *p* < .001 compared with control; 80 μg/mL EECU: 65.33%, *p* < .0001; 100 μg/mL EECU: 55.00%, *p* < .0001). Under phase‐contrast microscope, the phenotypic characteristics of EECU‐treated cells showed irregular cell outlines, decrease of cell density, and increase of detached cell (Figure 1b, top). To determine whether the cell growth inhibition by EECU was associated with apoptosis induction, we performed flow cytometric analysis and observation of nuclear morphology. The result of DAPI staining showed that the EECU‐treated cells observed were typical apoptotic nuclei, revealing nuclear fragmentation, destruction of cell membrane integrity, and chromatin condensation (Figure 1b, bottom). The results of the flow cytometric analysis showed that the percentage of annexin V^+^/PI^−^ cells and annexin V^+^/PI^+^ cells was markedly increased in EECU‐treated cells in a dose‐dependent manner (Figure 1c). In addition, the quantitative results of apoptotic cells showed that EECU induced significant apoptosis of B16F10 cells to 26.65% and 48.19% in 50 and 100 μg/mL, respectively (Figure 1d).

### 3.2 EECU increased mitochondrial dysfunction in B16F10 melanoma cells

**Figure 2:**
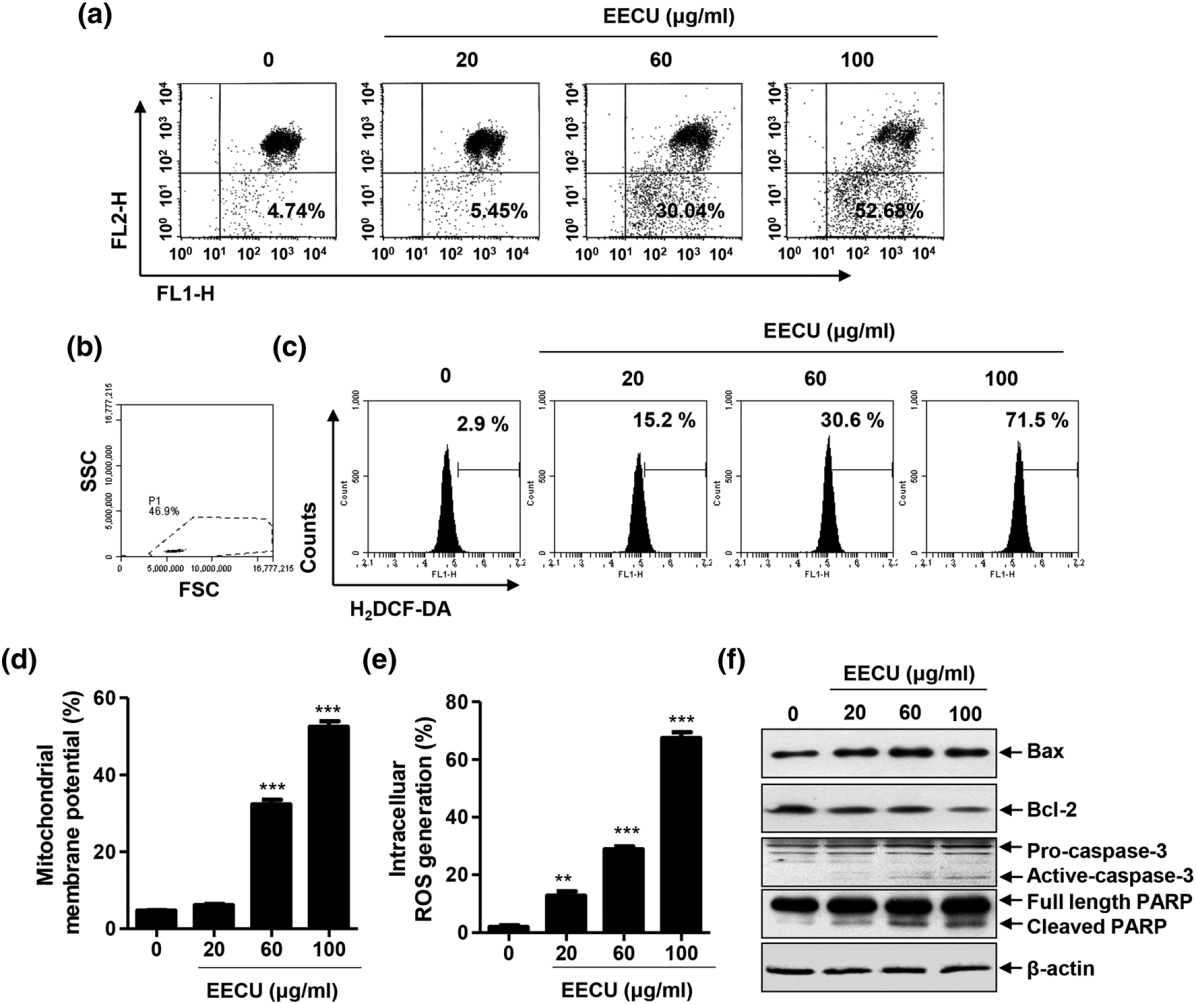
The effect of ethanol extracts of *Citrus unshiu* Marcow. fruits (EECU) on the mitochondrial dysfunction in B16F10 melanoma cells. (a) mitochondrial membrane potential (Δ*ψ*_m_) was assessed 24 hr after cells were treated with EECU of 20–100 μg/mL. The cells were stained with JC‐1 dye and then analyzed at 488/575 nm using a flow cytometer. Representative FL1/FL2 profiles with green/red fluorescence are shown. A plot of red fluorescence (FL2‐H) from living cells with intact mitochondrial membrane potential and green fluorescence (FLl‐H) from cells with loss of mitochondrial membrane potential was recorded. (b and c) Intracellular reactive oxygen species generation was measured by flow cytometry using DCF‐DA dye. The cells were treated with EECU for 30 min and were incubated with 10 μM DCF‐DA in the last 20 min of treatment. B16F10 cells were gated on the basis of forward‐scatter characteristics (FSC) and side‐scatter characteristic (SSC). (d and e) The quantitative data are expressed in the bar diagram as the mean ± *SD* (*n* = 3). The statistical analyses were conducted using analysis of variance (ANOVA‐Tukey's post hoc test) between groups. ^**^ *p* < .01 and ^***^ *p* < .001 when compared with control. (f) The expression of apoptosis‐related proteins in B16F10 melanoma cells treated with EECU. After the cells were incubated with EECU of 20–100 μg/mL for 24 hr, the expression of Bax, Bcl‐2, caspase‐3, and PARP was evaluated by Western blot analysis with whole cell lysates

We evaluated the effect of EECU on mitochondrial dysfunction in B16F10 cells. Mitochondrial dysfunction plays a critical role in intrinsic apoptosis pathway, which is involved in a change of the MMP (Δ*ψ*_m_) and production of ROS (Gross, McDonnell, & Korsmeyer, 1999). Therefore, we assessed the mitochondrial function using JC‐1 dye, an indicator of MMP (Δ*ψ*_m_), by a flow cytometer. The results showed that the loss of MMP (Δ*ψ*_m_) was significantly increased by EECU treatment compared with control in a concentration‐dependent manner (Figures 2a,c, *p* < .0001). In addition, we evaluated intracellular ROS generation using DCF‐DA probe. Figure 2c,e shows the results of flow cytometric analysis, which indicate that the ROS generation gradually increased according to the rise in concentration of EECU. Figure 2e suggests that the treatment of EECU led to the gradually up‐regulated expression of proapoptotic Bax and down‐regulated expression of antiapoptotic Bcl‐2, which are associated with mitochondrial‐mediated intrinsic pathway. In addition, EECU increased the expression of active caspase‐3 and induced the cleavage of PARP, one of the substrates of caspase‐3.

### 3.3 EECU reduced the motility of B16F10 melanoma cells via the inhibition of MMPs expression and activity

**Figure 3:**
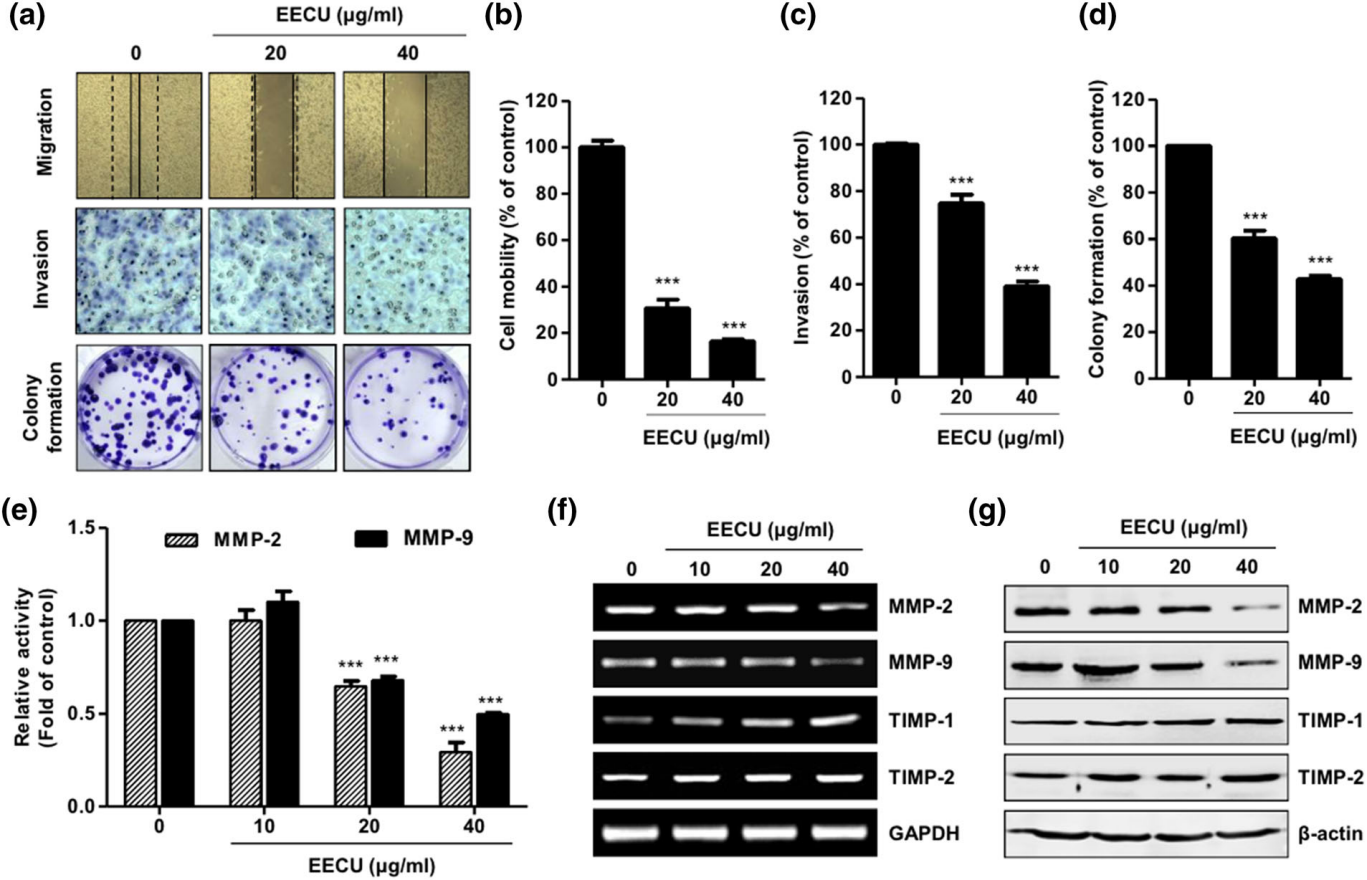
Ethanol extracts of *Citrus unshiu* Marcow. fruits (EECU) suppress the motility of B16F10 melanoma cells via the regulation of matrix metalloproteinases (MMPs) activity and expression. (a; top) Cell migration of B16F10 cells was assessed by wound healing assay at 24 hr after EECU treatment. Representative photographs are shown from three independent cell migration experiments. The dotted line indicates the baseline immediately at wound scratch, whereas the solid line indicates the migration at 24 hr after EECU treatment. (Middle) Cell invasion assay was evaluated using the trans‐well chamber system. B16F10 cells were placed in the upper chamber of trans‐well insert, and complement medium supplemented with EECU of 0, 20, and 40 μg/mL was added in the lower chamber, and then cells were incubated for 24 hr. (Bottom) B16F10 cells were exposed to EECU of 0, 20, and 40 μg/mL for 15 days, followed by colony formation assay. Cells were stained with 0.1% crystal violet solution, and visualized colonies were observed under microscope. (b) The mobility of B16F10 cells was calculated for EECU‐treated cells, as compared with the nontreated control cells for each experiment in different field. (c) The numbers of invading cells in EECU‐treated cells, as compared with the nontreated control cells, for each experiment. (d) Rates of colony formation were detected by microplate reader at 650 nm. The quantitative data are expressed in the bar diagram as the mean ± *SD* (*n* = 3). The statistical analyses were conducted using analysis of variance (ANOVA‐Tukey's post hoc test) between groups. ^***^ *p* < .0001 when compared to control. (e) Determination of MMP‐2 and MMP‐9 enzymatic activity by ELISA assay. Relative activities of MMP‐2 and MMP‐9 in EECU‐treated cells are indicated as ‐fold of control. The data are expressed as the mean ± *SD* (*n* = 3), and the statistical analyses were conducted using analysis of variance (ANOVA‐Tukey's post hoc test) between groups. ^***^ *p* < .0001 when compared with control. (f) mRNA expression of extracellular matrix remodeling‐related enzymes by EECU in B16F10 cells. At 24 hr after EECU treatment, total RNA was isolated from cells, and quantitative reverse transcription polymerase chain reaction analysis performed of MMP‐2, MMP‐9, TIMP‐1, and TIMP‐2 mRNA expression, using the indicated primers. GAPDH was used as the internal control. (g) Protein expression of ECM remodeling‐related enzymes by EECU in B16F10 cells. After the cells were incubated with EECU of 20–100 μg/mL for 24 hr, the cellular protein expression of MMP‐2, MMP‐9, TIMP‐1, and TIMP‐2 was measured by Western blot analysis with whole cell lysates [Colour figure can be viewed at http://wileyonlinelibrary.com]

To assess the effect of EECU on metastatic activity, we investigated the migration and invasion of B16F10 melanoma cells using wound scratch assay and trans‐well system, respectively. Figure 3a top panels show that EECU suppressed the closure rate of the scratch at 24 hr treatment, compared with the control B16F10 cells. In particular, the cell mobility was completely suppressed to 16.33% by 40 μg/mL of EECU (Figure 3b, *p* < .0001 vs. control). Moreover, EECU apparently decreased the invasion of B16F10 cells in dose‐dependent manner in trans‐well chamber assay (Figure 3a, middle panels). The invasive cells were repressed to 74.67% and 39.00% of control by 20 and 40 μg/mL of EECU, respectively (Figure 3c, *p* < .0001, compared with control). The results are consistent with the result of the wound scratch assay. Furthermore, we evaluated the effect of EECU on the colony formation that is a characteristic of tumor cells, and closely related to carcinogenesis (Hseu et al., 2012). As shown in the bottom panels of Figure 3a, the colony forming ability of B16F10 cells was markedly decreased by EECU relative to the control, in a concentration‐dependent manner. The colony formation was suppressed to 60.33% and 42.67% of control by 20 and 40 μg/mL of EECU, respectively (Figure 3d, *p* < .0001, compared with control). These findings suggest that the treatment of EECU decreased mobility and the colony formation of melanoma cells.

Because the degradation of extracellular matrix is an essential step in metastasis formation (Hofmann, Westphal, Van Muijen, & Ruiter, 2000), we investigated whether EECU regulates the activity and expression of matrix MMPs. Figure 3e indicates the activity of MMP‐2 substantially decreased to 0.29‐fold of control, and as well, 40 μg/mL of EECU significantly repressed the activity of MMP‐9 to 0.50‐fold of control (*p* < .0001). In addition, EECU effectively decreased the mRNA and protein expression of MMP‐2 and MMP‐9 in B16F10 cells (Figure 3f,g). In contrast, EECU dose‐dependently increased the mRNA and protein expression of TIMP‐1 and TIMP‐2 (Figure 3f,g). These data further demonstrate that EECU inhibited the mobility of the melanoma cells through the down‐regulation of MMPs and up‐regulation of TIMPs, and it may lead to the decrease of metastasis.

### 3.4 EECU suppressed the lung metastasis of B16F10 cells in C57BL/6 mice

**Figure 4:**
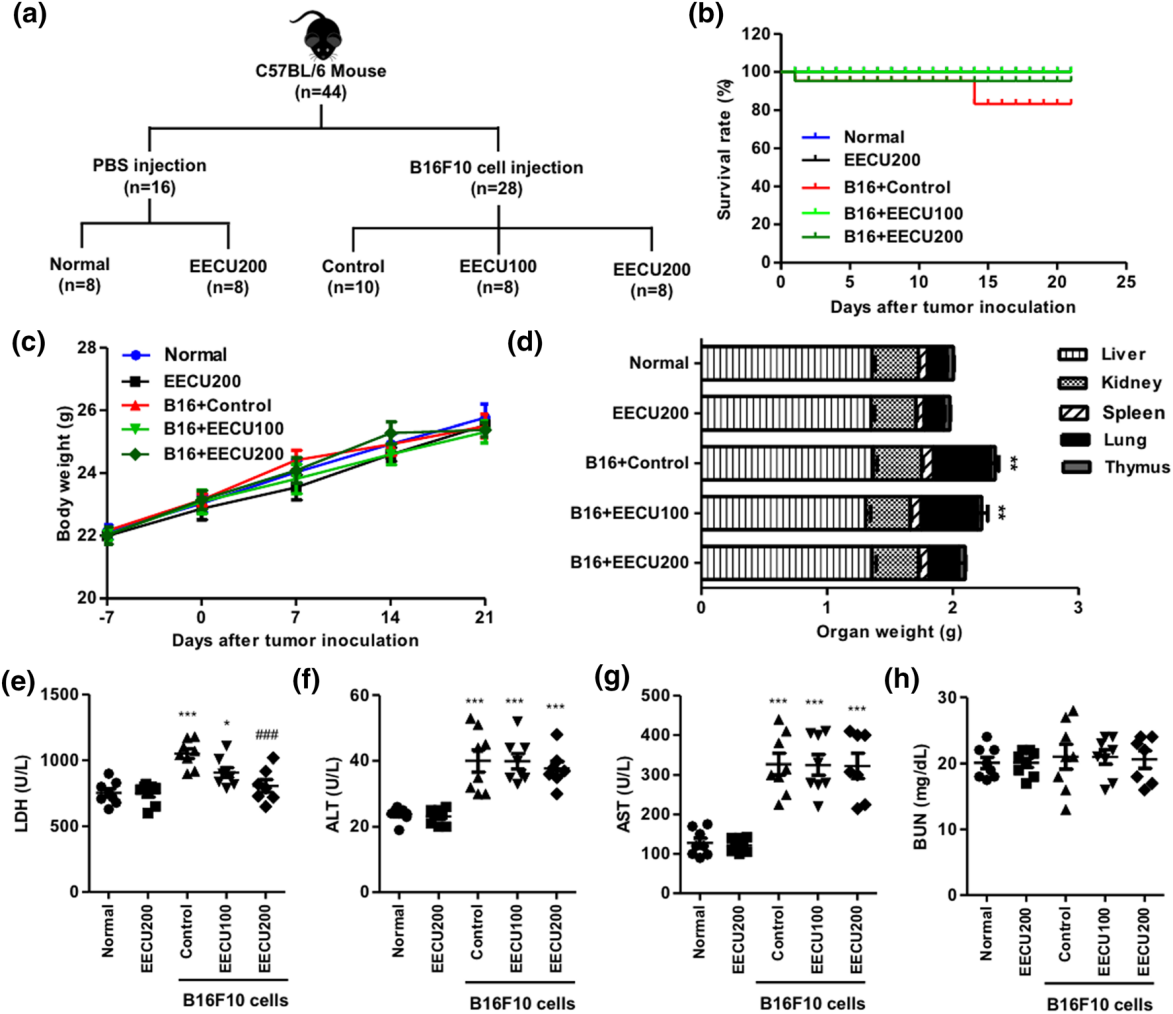
The effects of oral administration of ethanol extracts of *Citrus unshiu* Marcow. fruits (EECU) in B16F10 cells‐inoculated C57BL/6 mice. (a) Experimental design of in vivo study. For 28 mice, the mouse model of metastatic lung cancer was experimentally induced in 8 weeks old C57BL/6 mice by intravenous injections into the tail vein with 3 × 10^5^ B16F10 cells/100 μL phosphate‐buffered saline (PBS). At the same time, 16 mice were injected in the same area with PBS. After 1 day of tumor inoculation, B16F10 cell‐injected mice were randomly divided into three groups: the B16 + control group (*n* = 10, 100 μL of distilled water), the B16 + EECU 100 group (*n* = 8, 100 μL of EECU 100 mg/kg/day), and the B16 + EECU 200 group (*n* = 8, 100 μL of EECU 200 mg/kg/day). PBS‐injected 18 mice were also randomly divided into two groups: the normal group (*n* = 8, 100 μL of distilled water) and the EECU 200 group (*n* = 8, 200 μL of EECU 200 mg/kg/day). All treatments were administrated orally once per day in the morning for 21 days. (b) Kaplan‐Meier graph representing the cumulative survival of mice in the indicated treatment groups. The data shows the survival rate of the experimental period and were analyzed using Kaplan‐Meier survival analysis (*n* = 8–10 per group). (c) Body weight change of mice with the oral administration of EECU in B16F10‐induced metastatic lung cancer. Body weight was measured every 7 days. There were no differences in body weight between the groups. (d) Mice were sacrificed at Day 21 after B16F10 melanoma cells injection, and liver, kidney, spleen, lung, and thymus were immediately surgically excised and then measured the weight. The statistical analyses were conducted using analysis of variance (ANOVA‐Tukey's post hoc test) between groups. ^***^ *p* < .0001 when compared with normal group on weight of lung. (e) plasma lactate dehydrogenase (LDH), (f) alanine aminotransferase (ALT), and (g) aspartate aminotransferase (AST) activities and (h) blood urea nitrogen (BUN) levels after 21 days EECU treatment in B16F10‐induced metastatic lung cancer. The data are expressed as the mean ± *SD* (*n* = 7–8). The statistical analyses were conducted using analysis of variance (ANOVA‐Tukey's post hoc test) between groups. ^*^ *p* < .05 and ^***^ *p* < .001 when compared with normal group. ^###^ *p* < .001 when compared with B16F10 cell‐injected control group [Colour figure can be viewed at http://wileyonlinelibrary.com]

To investigate the effect of EECU on lung metastasis in vivo, the lung metastatic mouse model was induced by injection of B16F10 melanoma cells into the tail vein in C57BL/6 mice. A total of 44 mice were involved in this experiment. Twenty‐eight mice were injected with B16F10 cells, and 16 mice were injected with PBS as vehicle. From 1 day after, EECU 100 mg/kg, EECU 200 mg/kg, or distilled water were administrated orally once per day in the morning for 21 days (Figure 4a). One day after tumor cell inoculation, one animal died in the control group and one in the EECU 200 mg/kg group. In the control group, two of them died 14 days after tumor inoculation, and the survival rate on the 21 days was 83.33%. However, the survival rate was not significantly different between the control and EECU groups (Figure 4b). At Day 21, 100% of the normal mice and the EECU 200 mg/kg treated mice without B16F10 cell inoculation were still alive. The initial and final body weight did not differ between all the groups (Figure 4c). Mice were sacrificed at Day 21 of treatment, and their organs were surgically excised. As shown in Figure 4d, the weight of the liver, kidney, spleen, and thymus were not significantly different between all groups, exclusive of lung. The B16F10 cells‐injected control mice markedly induced lung hypertrophy (3.00‐fold of lung weight in normal, *p* < .001), whereas it was substantially decreased by EECU 200 mg/kg treatment. Additionally, we performed biochemical analysis of the LDH activity, hepatic, and renal function, including ALT, AST, and BUN. The result of analysis for plasma LDH activity, as the most consistent marker of the aggressive carcinogenesis (Chaube et al., 2015), proposed that the LDH activity was apparently increased to 1,050.25 U/L in B16F10 cells‐injected control mice (*p* < .0001 compared with normal mice). However, the LDH activity was meaningfully decreased by the administration of EECU 200 mg/kg (807.14 U/L, *p* < .0001 compared with control mice), and its activity was similar to that of the normal mice (Figure 4e). Meanwhile, we found that the activities of plasma ALT and AST were elevated in 40.00 and 326.88 U/L, respectively, in B16F10 cells‐injected control mice, whereas the activities of these enzymes were unchanged, following the administration of EECU (Figure 4f,g). In contrast, the levels of BUN showed no significant differences for all groups (Figure 4h). These results suggest that EECU not only reduced lung hypertrophy, but also LDH activity in B16F10‐inoculated mice.

**Figure 5:**
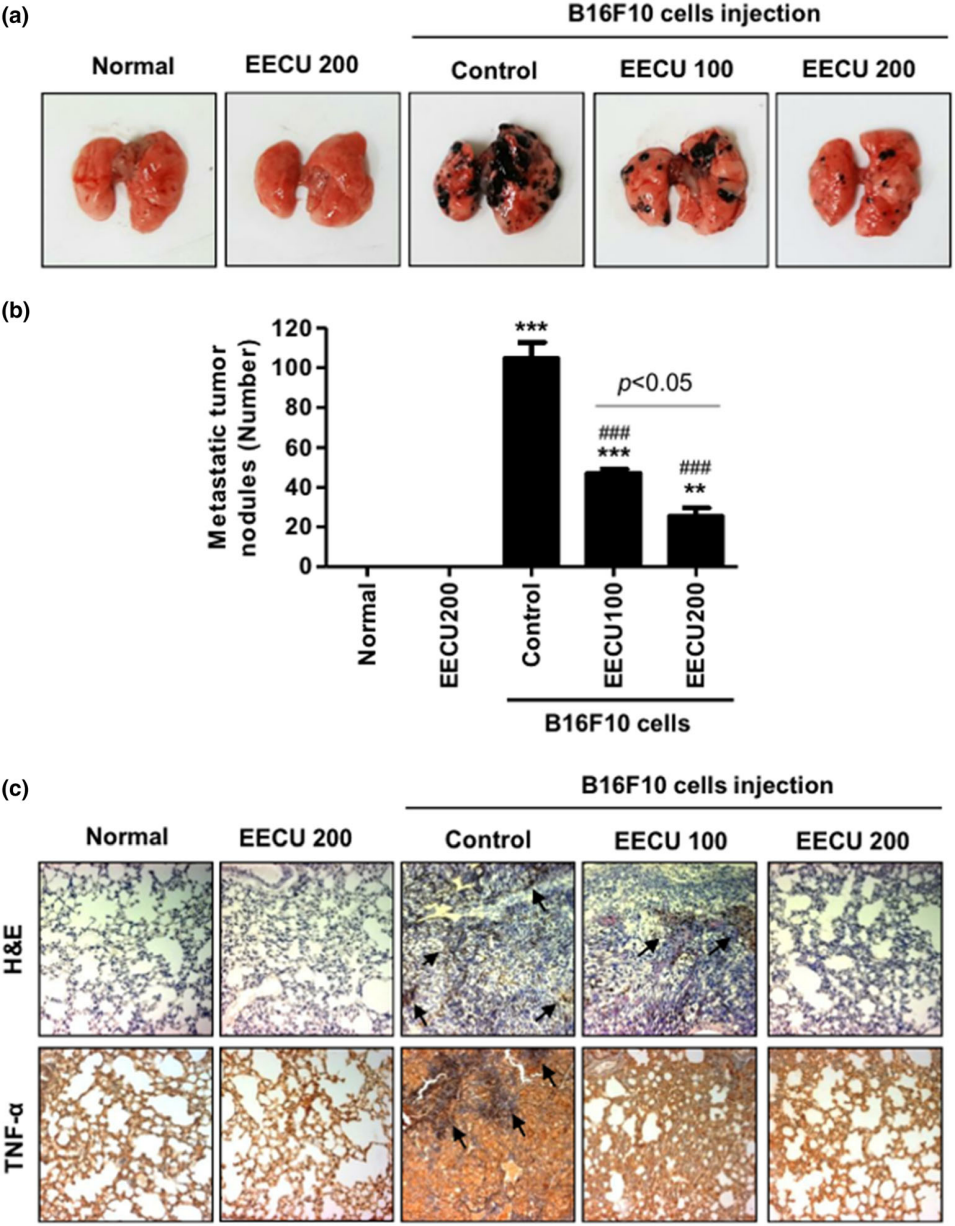
Orally administration of ethanol extracts of *Citrus unshiu* Marcow. fruits (EECU) suppressed B16F10 cells‐induced metastatic lung cancer. (a) Photographs of lung tissue at Day 21 after treatment. (b) The metastatic nodules were counted, and data presented as the mean ± *SD* (*n* = 7–8). The statistical analyses were conducted using analysis of variance (ANOVA‐Tukey's post hoc test) between groups. ^**^ *p* < .01 and ^***^ *p* < .001 when compared with normal group. ^###^ *p* < .001 when compared with B16F10 cell‐injected control group. (c; top) Immunopathological damage assessed by hematoxylin and eosin (H&E) staining of the lung sections. The images of the sections were photographed by microscope (Carl Zeiss). Black arrow indicates lung metastatic foci. Original magnification: 200×. (Bottom) The expression of tumor necrosis factor alpha (TNF‐α) of lung sections. Tumor tissues of lung metastasis were immunohistochemistry stained with TNF‐α. Black arrow indicates the TNF‐α‐expressed area shown in brown color, and marks the metastasis nodule. Original magnification: 200 × [Colour figure can be viewed at http://wileyonlinelibrary.com]

Next, we investigated the effect of EECU on the histopathological alteration of lung metastatic tissue following B16F10 inoculation. The number of metastatic tumor nodules in B16F10‐injected control mice was apparently increased in comparison with normal mice visually (Figure 5a), as well as numerically (Figure 5b, 105 ± 13, *p* < .0001 compared with normal mice). In contrast, the number of metastatic tumor nodules was significantly reduced by the oral administration of EECU in a dose‐dependent manner. Similar to the results from the count of metastatic tumor, the results of H&E staining also showed that brown‐spotted tumor cells were increased by B16F10 cells inoculation, whereas they were decreased by EECU administration (Figure 5c). In addition, we evaluated whether EECU could suppress lung inflammation in these mice. Figure 5c shows that TNF‐α of lung metastatic tissue was overexpressed in B16F10 cell inoculated mice but completely suppressed in EECU‐treated mice. These results clearly demonstrate that EECU could suppress lung metastasis and inflammation.

### 3.5 The phytochemical compositions of EECU

**Figure 6:**
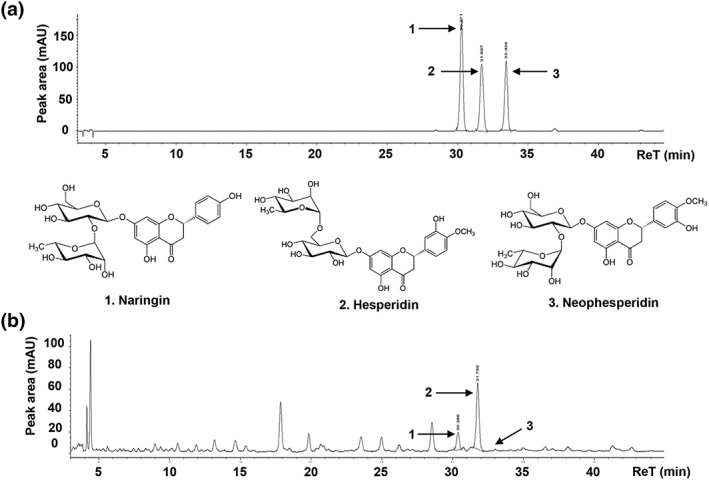
The phytochemical compound chromatogram from high‐performance liquid (HPL) analysis of ethanol extracts of *Citrus unshiu* Marcow. fruits (EECU). (a) Three reference components of naringin, hesperidin, and neohesperidin were analyzed using high‐performance liquid chromatography (HPLC). (b) The chromatograms from the HPLC analysis of EECU

To identify the bioactive compound of EECU, we further performed HPLC analysis. Figure 6a shows the chromatogram of reference compound, whereas Figure 6b shows the extracted compound HPLC chromatogram of EECU. The chromatograms indicate the presence of flavonoid compounds, namely naringin and hesperidin from EECU, and were detected at 30.32 and 31.72 of retention time, respectively. Quantitative analysis of these three compounds was conducted; the quantity of hesperidin of (5.61 ± 0.02) μg/mg was higher than of naringin of 2.15 ± 0.02 μg/mg (Table 1). However, neohesperidin, another reference flavonoid, was not detected in EECU.

## 4 DISCUSSION

Apoptosis is well‐known as programmed cell death and maintains a healthy balance between cell survival and cell death in an organism as an essential mechanism for maintaining cellular homeostasis (Bold, Termuhlen, & McConkey, 1997). Cancer occurs as a result of a series of genetic alterations, during which a normal cell is transformed into a malignant one that is involved in abnormal growth and the uncontrolled proliferation of cells (Hanahan & Weinberg, 2000). Hence, most chemotherapeutic drugs function by inducing apoptosis in malignant cells so the induction of apoptosis can be a major strategy of cancer therapy (Bold et al., 1997). Based on this situation, we have confirmed in previous studies that CU peel has anticancer efficacy via apoptosis, thus we investigated whether EECU ethanol extracts of CU peel have potential prevention against melanoma in vitro and in vivo. In the present study, we have verified that EECU inhibited cell growth in a dose‐dependent manner (Figure 1a). Additionally, EECU markedly increased the percentage of annexin V^+^/PI^−^ cells and annexin V^+^/PI^+^ cells (Figure 1c,d). Furthermore, EECU‐treated cells showed the typical morphological hallmarks of apoptotic cells, such as nuclear fragmentation, chromatin condensation, nucleus irregularity shape, and cell shrinkage (Figure 1b). These results suggest that the inhibition of cell growth by EECU is associated with the induction of apoptotic cell death in B16F10 melanoma cells and also corresponds with the results of our previous studies that EECU stimulated apoptosis in MCF‐7 breast cancer cells (Kim, HwangBo, et al., 2018) and T24 bladder cancer cells (Kim, Choi, et al., 2018). The intrinsic apoptosis signal pathway is initiated by irreparable genetic damage, oxidative stress, high concentration of cytosolic Ca^2+^, and hypoxia, which converge at the mitochondria (Karp, 2008). This pathway is the result of a mitochondrial dysfunction that includes loss of MMP (Δ*ψ*_m_), production of ROS, opening of the permeability transition pore, and release of cytochrome *c*, which is closely regulated by Bcl‐2 family (Gross et al., 1999; Wong, 2011). There are two main groups of the Bcl‐2 proteins, namely the antiapoptotic proteins (Bcl‐2, Bcl‐XL, Bcl‐W, etc.) and the proapoptotic proteins (Bad, Bax, Bak, Bid, Bik, etc.; Reed, 1997). The release of cytochrome *c* from the mitochondria to the cytoplasm induces caspase‐3 via the formation of apoptosome, which consists of caspase‐9, Apaf‐1, and cytochrome *c* (Reed, 1997). Caspase‐3 converged both intrinsic and death receptor‐initiated extrinsic pathways and degrades various substrate proteins, such as PARP (Decker & Muller, 2002). Downstream caspases induce the cleavage of cytoskeletal proteins, protein kinases, and DNA repair proteins, thus leading to alteration of the cytoskeleton and cell cycle, which contribute to the morphological changes in apoptosis (Ghobrial, Witzig, & Adjei, 2005; Wong, 2011). In the present study, we investigate whether apoptosis by EECU is associated with mitochondria dysfunction. Our results show that EECU significantly increased MMP (Δ*ψ*_m_) loss, as well as intracellular ROS generation, in B16F10 melanoma cells (Figures 2A–E). Moreover, EECU gradually up‐regulated the expression of proapoptotic Bax and down‐regulated the expression of antiapoptotic Bcl‐2. Additionally, EECU induced the activation of caspase‐3 and the cleavage of PARP (Figure 2F). These results demonstrate that EECU induced apoptosis through a mitochondria‐mediated intrinsic pathway in B16F10 melanoma cells.

Melanoma is a particularly aggressive skin cancer with a high capacity for invasion and metastasis, and resistance to cytotoxic antitumor drugs. This is estimated to be because melanocytes originate from highly motile cells that have enhanced survival properties (Gray‐Schopfer et al., 2007). Metastasis is caused by the movement of cancer cells from the primary tumor to target organs, thus blocking cancer cell migration and invasion, which are most important for the treatment of melanoma (Bhatia et al., 2009). Herein, we found EECU suppressed the migration and invasion of B16F10 cells in a dose‐dependent manner through the results of wound scratch assay and trans‐well assay. In addition, we confirmed EECU also inhibited anchorage‐dependent colony formation that is a characteristic of tumor cells and closely related to carcinogenesis (Figure 3a,b; Hseu et al., 2012). These results indicate that EECU blocks the migration and invasion, a key step of the metastasis of melanoma, as well as the establishment of anchorage‐dependent colony formation from a single cell. Degradation and remodeling of the extracellular matrix and basement membranes are essential steps in the metastasis of melanoma. These processes are mediated by proteolytic enzymes, such as MMPs (MMP‐1, ‐2, ‐9, ‐13, and MT1‐MMP) and their tissue inhibitors (TIMPs; TIMP‐1, ‐2, and ‐3), and the regulation of their expression and/or activation on invasion and migration in many types of tumors has been widely reviewed in vitro and in vivo (Hofmann et al., 2000). In particular, MMP‐2 and MMP‐9 are well‐known to induce cancer progression and the metastasis of melanoma through the degradation of Type IV collagen, which is the major component of the basement membrane (Hofmann et al., 1999). In this regard, we demonstrated that EECU markedly decreased the activity of MMP‐2 and MMP‐9 (Figure 3E). Furthermore, our results proved that EECU induced the down‐regulated expression of MMP‐2 and MMP‐9 as well as the up‐regulated expression of TIMP‐1 and TIMP‐2 in mRNA and protein levels (Figure 3f,g). Based on these findings, we suggest that EECU promotes an increase of TIMP/MMPs ratio as a critical factor in the regulation of the motility of melanoma cells, which may subsequently lead to the suppression of cell migration and invasion associated metastasis. Following that, we have reconfirmed the efficacy of EECU in the suppression of metastasis in melanoma‐inoculated mice. Interestingly, B16F10 cell has been metastasized specifically to the lung following the injection into the tail vein, and most of the cells have been found in the pulmonary tissue (Fidler, 1973). Therefore, the murine B16F10 melanoma is most accepted as a useful model for metastatic lung tumor: its application having been used to assess the metastatic mechanisms of melanoma and the development of anticancer therapies (Fidler, 1973; Giavazzi & Decio, 2014). Numerous studies reported the experimental lung or pulmonary metastasis model by B16F10 inoculation in C57BL/6 mice and investigated the efficacy of new anticancer drugs and potential phytochemicals in this murine model (Gautam, Densmore, & Waldrep, 2000; Pal et al., 2016; Siddikuzzaman & Grace, 2012). In the present study, we identified that B16F10 cells‐inoculated mice have induced lung hypertrophy and increase of the number and expression of metastatic tumor nodules in lung tissue, whereas these were significantly decreased by the oral administration of EECU (Figures 4D and 5). Additionally, the oral administration of EECU reduced serum LDH activity as the most consistent marker of the aggressive carcinogenesis (Chaube et al., 2015), without weight loss, hepatotoxicity, or nephrotoxicity (Figure 4c,e–h). These results suggest that EECU can be a considerably safe therapeutic medicinal plant for metastatic melanoma through its low toxicity and efficacy in the suppression of metastasis. Moreover, our study also provided that B16F10 inoculation promoted the overexpression of TNF‐α in the metastatic region, but was suppressed by EECU (Figure 5c). It is well‐known that inflammation plays principal roles at different stages of tumor development, including initiation, promotion, malignant conversion, invasion, and metastasis (Grivennikov, Greten, & Karin, 2010). Rayes et al. demonstrated that lung inflammation promotes metastasis (El Rayes et al., 2015), and Yu et al. reported proinflammatory cytokines, including TNF‐α, IL‐1, and IL‐6, accelerate MMPs expression, invasiveness, and metastasis (Yu, Kortylewski, & Pardoll, 2000). In this respect, our results provide the possibility of EECU suppressing inflammation, a hallmark of cancer contributing to tumor development, and may lead to the inhibition of metastasis in lung cancer.

The bioactive compounds of CU peel are well‐known to contain flavonoids, such as naringin, hesperidin, and nobiletin, and the plant cell barrier component and its anticancer effect are also well known (Jin et al., 2013; Kim et al., 2015; Park et al., 2018; Shin, Park, & Shin, 2018). Our previous study reported that water extract of CU peel contained the reference flavonoids (i.e., naringin, hesperidin, and neohesperidin) and had anticancer potential (Kim, Bo, et al., 2018). In common with previous study, in the present study, we identified that EECU contains bioactive flavonoids, including naringin and hesperidin (Figure 6 and Table 1). Recently, numerous studies reported that naringin and hesperidin have the antimetastatic activity. Aroui et al. reported that naringin suppress cell invasion and adhesion of human glioblastoma U87 cells and U251 cells (Aroui et al., 2015, 2016). In addition, naringin have antimetastatic activity in human prostate cancer DU145 cells (Erdogan, Doganlar, Doganlar, & Turkekul, 2018). Furthermore, it is reported that hesperidin has suppressive effect of cell mobility in nonsmall cell lung cancer A549 cells (Xia et al., 2018) and human osteosarcoma MG‐63 cells (Du et al., 2018). Yu et al. also demonstrated that hesperin attenuated the epithelial to mesenchymal transition in A549 cells (Yu, Li, Ren, & Shen, 2016). Based on these previously studies, the anticancer effect of EECU in melanoma cells and metastatic lung cancer is believed to be attributed to various bioactive compounds, including flavonoids from EECU.

In summary, EECU inhibited cell growth through the induction of mitochondria‐mediated intrinsic apoptosis pathway in B16F10 melanoma cells. EECU also promoted down‐regulation of MMPs activity and expression, which is a critical factor in the regulation of the motility of melanoma cells, and may subsequently lead to the suppression of cell migration and invasion‐associated metastasis. In the metastatic lung cancer mouse, EECU inhibited lung hypertrophy and metastatic tumor nodule, as well as inflammation in lung tissue without toxicity. When given orally administration, EECU has anti‐inflammatory and antimetastatic activities, which are dose‐dependent over a range of 100~200 mg/kg. In conclusion, although further studies are necessary to assess the potential clinical use of this plant, or its extract or active principles, our finding suggests the inhibitory effect of EECU on the metastasis of melanoma, and EECU can be considered as a potential therapeutic phytomedicine for melanoma.

### CONFLICT OF INTERESTS

The authors declare that there are no conflicts of interest.

## FUNDING INFORMATION

This research was supported by Basic Science Research Program through the National Research Foundation of Korea (NRF) grant funded by the Korea government (2018R1A2B2005705) and the International Science and Business Belt Program through the Ministry of Science, ICT and Future Planning (2017K000490).
